# Descriptive study of enteric zoonoses in Ontario, Canada, from 2010 – 2012

**DOI:** 10.1186/s12889-017-4135-9

**Published:** 2017-02-21

**Authors:** Yvonne Whitfield, Karen Johnson, Leigh Hobbs, Dean Middleton, Badal Dhar, Linda Vrbova

**Affiliations:** 1 Public Health Ontario, 480 University Avenue, Toronto, ON M5G 1V2 Canada; 2 The Public Health Agency of Canada, Guelph, ON Canada

**Keywords:** Enteric, Zoonoses, Exposures, Animals, Illness

## Abstract

**Background:**

Contact with animals and their environment has long been recognized as an important source of enteric zoonoses. However, there are limited data available on the burden of illness associated with specific types of animals in Canada. This study describes the overall burden of enteric zoonoses in Ontario, Canada from 2010 to 2012.

**Methods:**

Confirmed cases of seven enteric zoonotic diseases (campylobacteriosis, cryptosporidiosis, giardiasis, listeriosis, salmonellosis, verotoxin-producing *E. coli* (VTEC) infection, and yersiniosis) with episode dates from 2010 to 2012 were extracted from the integrated Public Health Information System (iPHIS). Reported exposures were categorized as animal contact, foodborne, waterborne and ‘other’, with animal contact grouped into nine sub-categories based on the type of animal or transmission setting. Overall incidence rates and proportions by animal exposure categories, age and sex-specific incidence rates and hospitalization and death proportions were calculated and sex proportions compared.

**Results:**

Our study found that approximately 26% of the enteric pathogens assessed during the 2010 to 2012 period reported contact with animals and their environments as the mode of transmission. Of enteric disease cases reporting animal contact, farm exposures were reported for 51.3%, dog or cat exposures for 26.3%, and reptile or amphibian exposures for 8.9%.

**Conclusions:**

Contact with animals was reported more frequently during the period 2010 to 2012 in comparison to the period 1997 to 2003 when 6% or less of enteric cases were associated with animal contact. Public health professionals, stakeholders associated with animals and their related industries (e.g., pet treats, mobile zoos, abattoirs), and the public should recognize that animal contact is an important source of enteric illnesses in order to take measures to reduce the burden of illness from animal sources.

## Background

Enteric diseases are a significant contributor to the overall burden of reportable illnesses in Ontario, accounting for approximately 9500 cases or 15% of reportable diseases that occur in the province [1]. Among enteric diseases in Ontario, *Campylobacter*, *Salmonella* and *Giardia* are the leading causes of infection [2]. It is estimated that each reported case of enteric disease in Ontario represents 105 to 1,389 infectious gastroenteritis cases that are underreported [3]. Consumption of contaminated food and water; contact with animals and their environment, and person to person contact constitute important sources of these infections [2]. In Ontario, estimates of enteric illness reported from exposures to animals vary, ranging from 5.8% for the years 1997 to 2001, 1.0% in 2003, and 19.8% for the years 2007 to 2009 [2, 4, 5]. A similar study in the USA in 2012 estimated that animal contact was responsible for 14% of enteric illnesses [6]. These studies did not provide any detail on the animal exposures associated with enteric infections beyond the percent attribution. For example, none have described the species of animals involved. This gap in knowledge has implications with respect to the types of prevention and control measures that could be implemented by public health authorities, industry and other stakeholders. The purpose of this study is to describe the overall burden of enteric zoonoses in Ontario from 2010 to 2012 in order to improve our understanding of enteric zoonoses and inform prevention and control measures in this area.

## Methods

### Data sources

Data for this study were obtained from Ontario Ministry of Health and Long-Term Care’s integrated Public Health Information System (iPHIS) database, and were extracted by Public Health Ontario on 2013/09/06. Pursuant to the *Health Protection and Promotion Act*, public health units in Ontario follow up with cases of reportable diseases to provide case management services and to identify possible sources of disease acquisition (i.e., exposures) [7]. Information on case demographics, relevant exposures and outcomes (i.e., hospitalization and death) are reported to the province through iPHIS. Confirmed cases of seven enteric zoonotic diseases (campylobacteriosis, cryptosporidiosis, giardiasis, listeriosis, salmonellosis, verotoxin-producing *E. coli* (VTEC) infection, and yersiniosis) with episode dates from January 1, 2010 to December 31, 2012 were obtained from iPHIS. Episode dates are defined as the earliest of symptom onset, specimen collection, or reported date. Surveillance case definitions for these diseases are available online [8]. Population data used for the calculation of incidence rates were obtained from Statistics Canada via IntelliHealth ONTARIO [9].

### Mode of transmission - all cases

Exposures reported in iPHIS are assigned by case investigators and are defined as the most likely source(s) of illness. These exposures were categorized by mode of transmission as follows: animal contact, foodborne, waterborne, and other, with the ‘other’ category comprising person-to-person transmission and transmission via fomites other than food and water. Exposures that did not fit into these transmission categories were categorized as ‘unclassifiable’. These exposures along with exposures reported as ‘unknown’ and exposures that were missing were removed from analyses as appropriate.

### Mode of transmission - animal contact

Cases reporting animal contact were further categorized into nine sub-categories based on the type of animal and the transmission setting: ‘farm’ (i.e., direct or indirect contact with livestock on a farm and/or occupational exposures at abattoirs and food processing plants); ‘zoo’ (i.e., direct or indirect contact with animals at a zoo); ‘dog or cat’; ‘reptiles or amphibians’; ‘wild animals’; ‘rodents’; ‘exotic animals’(e.g., direct or indirect contact with ornamental fish, rabbits and pet birds); ‘pet food’ and ‘unspecified’ (i.e., an exposure where the type of animal or setting was not reported). These sub-categories were based on a modified version of Hale’s definition of animal contact which was defined as direct and/or indirect contact with live animals of any type, including food-producing animals at the point of slaughter but not contact with meat after processing. Indirect contact included animal feces, fluids or their environment [6]. To avoid over-estimation of the proportion of cases attributed to animal exposures, we excluded cases that reported both animal contact and at least one of the other defined modes of transmission, resulting in an “animal contact only” category.

### Animal contact only - other variables

Hospitalization status defined as having at least one hospital admission date and death reported as a contributing or underlying cause were included in analyses as measures of illness severity. Age was categorized as follows: <1, 1-4, 5-14, 15-24, 25-44, 45-64 and ≥65 years.

### Animal contact only - descriptive and statistical analyses

The overall, age and sex-specific incidence rates, were calculated for each disease using the 2010, 2011, and 2012 Ontario populations. The three year sex-specific incidence rate was calculated by adding the number of male and female cases in 2010, 2011 and 2012, respectively, and dividing by three. This number was divided by the mean population of Ontario for the three years for each gender, and multiplied by 100,000. The three year age-specific incidence rate was calculated by adding the number of male and female cases for each age group in 2010, 2011 and 2012, respectively, and dividing by three. This number was divided by the mean population of Ontario for the three years for each age group, and multiplied by 100,000.

Disease-specific proportions for each mode of transmission and by outcome (i.e., hospitalization and death) were also calculated. For certain analyses, cases were excluded if they had a reported history of travel outside of Ontario or if they had exposures that were missing, unclassifiable or reported as ‘unknown’. Descriptive analyses were performed using SAS 9.3 (SAS Institute Inc., Cary, NC, USA) and figures were constructed using Microsoft Excel (Microsoft Corporation, version 2010, Redmond, WA, USA). The exact hypothesis test was used to test whether the proportion of sexes was equal using the binomial distribution in Stata (StataCorp. 2013. Stata Statistical Software: Release 13.1. College Station, TX: StataCorp). The Public Health Ontario Ethics Review Board determined that research ethics review was not required for conducting this study.

## Results

### Mode of transmission – all cases

**Fig. 1:**
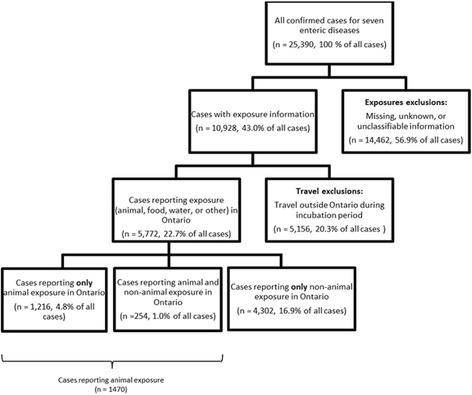
Number of confirmed cases of seven reportable enteric diseases in each stage of selection for final analytic sample of cases, Ontario, 2010 to 2012

From 2010 to 2012, 25,390 cases of campylobacteriosis, cryptosporidiosis, giardiasis, listeriosis, salmonellosis, VTEC infection and yersiniosis were reported in Ontario (Fig. 1). Of these, cases with missing, unknown or unclassifiable exposure information were removed, leaving 10,928 cases that had at least one exposure reported. Travel-associated cases were subsequently removed, leaving 5,772 cases with domestically acquired exposures.

Foodborne was the most frequently reported mode of transmission. A total of 3,358 (58.2%) foodborne exposures were reported to have occurred among the 5,772 cases. Animal contact was the second most frequent mode of transmission with 1,470 (25.5%) exposures, followed by waterborne with 734 (12.7%), and ‘other’ at 619 (10.7%). Of the animal contact cases, there were 1,216 (21.1%) cases that were assessed to be animal contact only and 254 (4.4%) that were assessed to have had animal contact as well as another mode of transmission (Fig. 1).

### Mode of transmission - cases reporting animal contact only

Of the 1,216 cases where animal contact was the sole exposure, 624 (51.3%) farm exposures were identified. Dog or cat exposure was the second most frequently identified exposure (320, 26.3%), followed by reptile or amphibian exposures (108, 8.9%) (Table 1). There were 11 exposures due to amphibians reported in the reptile or amphibian sub-category. Campylobacteriosis was identified as the most common enteric infection in those who had solely animal exposure 685 of 1,216 cases (56.3%)). Salmonellosis was the second most frequently identified enteric infection (301 cases, 24.8%), followed by cryptosporidiosis (113 cases, 9.3%). For salmonellosis, S. Enteritidis and S. Typhimurium were each responsible for 17.9% of cases followed by S. Heidelberg with 11.3% of cases.

**Table 1:** Enteric disease by animal contact only sub-category, Ontario, 2010 to 2012

Disease	Farm	Dog or cat	Reptiles or Amphibians	Zoo	Wild	Exotic	Rodent	Pet food	Unspecified^a^	Total cases reporting only animal exposure^bc^	Total cases reporting exposure information
	*n* (%)	*n* (%)	*n* (%)	*n* (%)	*n* (%)	*n* (%)	*n* (%)	*n* (%)	*n* (%)	*n*	*n*
Campylobacteriosis	398 (58.1)	207 (30.2)	1 (0.1)	8 (1.2)	16 (2.3)	10 (1.5)	6 (0.9)	7 (1.0)	81 (11.8)	685	2414
Salmonellosis	85 (28.2)	66 (21.9)	107 (35.5)	24 (8.0)	14 (4.7)	9 (3.0)	12 (4.0)	12 (4.0)	28 (9.3)	301	2058
Cryptosporidiosis	87 (77.0)	7 (6.2)	0 (0.0)	5 (4.4)	2 (1.8)	1 (0.9)	1 (0.9)	0 (0.0)	12 (10.6)	113	608
Giardiasis	21 (30.0)	33 (47.1)	0 (0.0)	2 (2.9)	8 (11.4)	4 (5.7)	2 (2.9)	0 (0.0)	8 (11.4)	70	291
Verotoxin-producing *E. coli* infection	33 (84.6)	4 (10.3)	0 (0.0)	5 (12.8)	0 (0.0)	1 (2.6)	0 (0.0)	1 (2.6)	1 (2.6)	39	242
Yersiniosis	0 (0.0)	3 (37.5)	0 (0.0)	1 (12.5)	1 (12.5)	0 (0.0)	1 (12.5)	0 (0.0)	2 (25.0)	8	92
Listeriosis	0 (0.0)	0 (0.0)	0 (0.0)	0 (0.0)	0 (0.0)	0 (0.0)	0 (0.0)	0 (0.0)	0 (0.0)	0	67
Total	624 (51.3)	320 (26.3)	108 (8.9)	45 (3.7)	41 (3.4)	25 (2.1)	22 (1.8)	20 (1.6)	132 (10.9)	1216	5772

^a^Includes exposures where the type of animal or setting was not reported

^b^For each disease and overall, the total number of cases reporting exposure information over the three-year period was used as the denominator to calculate proportions

^c^Cases can report multiple animal contact only subcategories. As a result proportions may not add to 100%

### Descriptive and statistical analyses - cases reporting animal contact only

The 1,216 cases reporting animal contact only corresponded to a three-year average incidence rate of 3.0 cases per 100,000 population. The three-year average sex-specific incidence rate was higher for males compared to females (3.4 vs. 2.6 cases per 100,000 population). This difference is driven largely by the significantly higher proportion of male (61%) to female (39%) campylobacteriosis cases (*p* < 0.0001), since the proportions were similar (i.e., there were not statistically significant differences) for all of the other studied diseases.

**Fig. 2:**
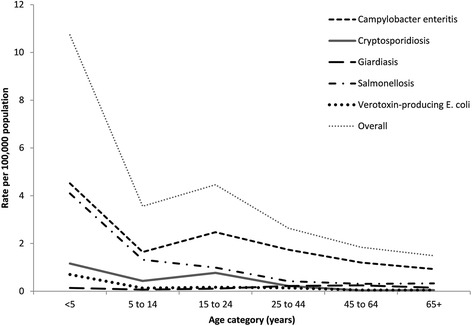
Three-year average age-specific incidence rate by disease^*^ for cases reporting animal contact only, Ontario, 2010 to 2012

Overall, the highest three-year average rates of enteric disease were reported among children under 5 years of age (10.7 cases per 100,000 population) and among persons aged 15 to 24 years (4.5 cases per 100,000 population). While similar age-specific trends were observed for the studied diseases, rates for children under 5 years were highest for campylobacteriosis and salmonellosis at 4.5 and 4.1 cases per 100,000 population, respectively (Fig. 2). Over the three year period, a total of 97 cases reported being hospitalized (97/1,216 = 8.0%), with the highest proportion of hospitalizations reported among VTEC infection (11/39 = 28.2%) and cryptosporidiosis cases (16/113 = 14.2%). Death was reported for one case of salmonellosis.

## Discussion

### Mode of transmission – all cases

Foodborne was the most frequently reported mode of transmission for the enteric pathogens in this study covering the 2010 to 2012 period. The 58% foodborne cases reported during this period was less than the 74% of foodborne cases reported from 1997 to 2001 and the 73% of cases in 2003, but is similar to the 54% of cases identified from 2007 to 2009 in Ontario [2, 4, 5]. It appears that the percent of foodborne transmission may be lower in the period 2007 to 2012 compared to 1997 to 2003.

Animal contact was the second most frequent mode of transmission identified. Approximately one in four enteric illness cases were identified as having their illness caused by contact with an animal. In this study, other than foodborne transmission, animal contact was identified as frequently as the other modes of transmission (i.e., water and other) combined. In Ontario, previous studies reported animal contact as 6% in 1997 to 2001, 1% in 2003, and 20% in 2007 to 2009 [2, 4, 5]. The percent of animal contact transmission was higher in the period 2007 to 2012 compared to the period 1997 to 2003.

### Mode of transmission - cases reporting animal contact only

Farm exposures were reported for the majority of cases for the animal contact only sub-category. A systematic review and meta-analysis of 38 case-control studies showed that direct contact with farm animals and abattoirs was an important risk factor for campylobacteriosis [10]. In industrialized countries, most cases of campylobacteriosis have been attributed to animal reservoirs such as cattle and chicken, with attribution proportions ranging from 35-66% for cattle and 21-57% for chicken [10]. Our finding of on-farm exposure being an important pathway for cryptosporidiosis is also supported by studies that have demonstrated associations with farm visits and contact with cattle [11]. Expert elicitation surveys in Canada have attributed 13-23% of human cryptosporidiosis illness to direct animal contact, consistent with a US study that found this proportion to be 16% [12]. For VTEC infection, attribution studies have identified animal contact as the source of 10-28% of reported illnesses, with farm visits and living in a rural area independently shown to be important pathways for animal contact [13]. Given that less than 2% of the Ontario population lives on a farm and very few people visit farms, we suggest that farm exposures are over-represented in the findings [14]. This finding reinforces the need for a one-health approach wherein livestock workers, public health and veterinary health professionals implement strategies to reduce the risk of disease transmission on farms and in other occupational settings such as abattoirs.

Dog or cat exposure was reported for approximately one in four cases in the animal contact only sub-category. In Ontario, it is estimated that 68% of households own one or more dogs and 48% own one or more cats [15], suggesting that dog or cat contact is under-represented in our findings, or that a minority of these animals transmit disease. This is consistent with one study which showed that contact with dogs or cats did not constitute a major zoonotic risk for healthy individuals [16]. Thus, the observed proportion attributable to contact with dogs or cats may be reflective of high rate of dog or cat ownership rather than the true risk of these animals transmitting enteric pathogens such as *Salmonella*, *Giardia* and *Campylobacter*. Further, some of the risk of disease transmission may not be from the dogs or cats themselves but from pet treats (e.g., dog biscuits, pig ears, rawhide chews) that have been shown to be contaminated with various enteric pathogens such as *Salmonella* and *Campylobacter* and have been associated with illness in humans [17–19].

Almost one in ten cases identified exposures to reptiles or amphibians in the animal only sub-category, although only a small percentage of amphibian exposures were identified. The link between reptile contact and human salmonellosis has been clearly established, with attributable proportions ranging from 4-6% for sporadic salmonellosis cases [20, 21]. In our study, salmonellosis was almost exclusively the disease resulting from contact with reptiles or amphibians. Further, reptile or amphibian contact accounted for more than 1/3 of animal-associated salmonellosis cases overall. In Canada and the US, household contact with reptiles ranges from 5-6% [15]. It is also estimated that one percent of Canadians own [22] these animals. Our finding suggests that reptiles are over-represented as a cause of salmonellosis in Ontario. The serotypes associated with illness were *S.* Enteritidis, *S*. Typhimurium and *S*. Heidelberg, serotypes not traditionally associated with reptiles. This finding corroborates those of Whitten et al. who also found that reptile-associated salmonellosis was more frequently attributed to serotypes not considered to be associated with reptiles such as *S.* Enteritidis, *S*. Typhimurium and *S*. Bareilly [21]. Reptile-associated salmonellosis has been described as an emerging zoonosis among young children who are generally more susceptible to severe illness [20, 23]. The increasing popularity of mobile zoos, zoo-themed parties and school-based activities that involve reptiles and other exotic animals presents an increase in risk of exposure, particularly among susceptible populations. The U.S. Centers for Disease Control and Prevention and the Public Health Agency of Canada currently advise that children less than 5 years of age and immunocompromised individuals should avoid contact with reptiles [24, 25]. Educators and daycare operators will also need to carefully assess the risks versus the benefits of any decision to allow children under the age of five to be exposed to reptiles.

### Strengths and limitations

The main strength of our study is our use of routinely-collected reportable disease data which is population-based. These data complement the literature in this area that largely focuses on outbreaks of enteric disease transmitted via animals [26–32]. The reporting of sporadic enteric disease cases provides more complete epidemiologic findings than outbreak-focused studies which individually, or collectively in review studies, only report the source of respective outbreaks.

There were several limitations of the data used for this study. First, 14,462 (57.0%) of the 25,390 records available were excluded from analysis because of missing, unknown or unclassifiable information. Second, recall bias most likely occurred because cases were often interviewed two to three weeks after their onset of symptoms. The ability of cases to recall all relevant exposures after this period of time undoubtedly presented challenges. Third, it is difficult to identify the specific cause of the illness in sporadic case investigations. The association between exposure and illness was not usually corroborated by laboratory analyses of clinical specimens and/or environmental samples, or other analytic studies. As a result, the reported exposures do not necessarily represent a causal relationship with illness. Finally, the exposures identified were based on the case investigator’s best assessment of the potential source(s) of the case’s illness. The investigator’s interpretation would be influenced by their understanding of the various potential sources of the pathogen.

## Conclusions

Our findings suggest that contact with animals and their environment is an important source of enteric zoonoses. Approximately 26% of enteric diseases were associated with animal contact for the period 2010 to 2012. This is likely of greater public health significance than was previously understood in Ontario. Compared to the period 1997 to 2003 when 6% or less of enteric cases were associated with animal contact, the percent of animal contact transmission identified was higher in the period 2010 to 2012. Farm exposure was reported the most frequently, followed by contact with dogs or cats, and reptiles or amphibians. Public health professionals, stakeholders associated with animals and their related industries (e.g., pet treats, mobile zoos, abattoirs), and the public should recognize that animal contact is an important source of enteric illnesses in order to take measures to reduce the burden of illness from animal sources. This study is the first to examine the associations between animal exposures and enteric illnesses in Ontario. Future studies that examine causation will provide more definitive evidence to support the burden of illness associated with animals.
